# Role of Live-Duck Movement Networks in Transmission of Avian Influenza, France, 2016–2017

**DOI:** 10.3201/eid2603.190412

**Published:** 2020-03

**Authors:** Claire Guinat, Benoit Durand, Timothee Vergne, Tifenn Corre, Séverine Rautureau, Axelle Scoizec, Sophie Lebouquin-Leneveu, Jean-Luc Guérin, Mathilde C. Paul

**Affiliations:** École Nationale Vétérinaire de Toulouse, Toulouse, France (C. Guinat, T. Vergne, T. Corre, J.-L. Guérin, M.C. Paul);; Institut National de Recherche pour l’Agriculture, l’Alimentation et l’Environnement, Toulouse (C. Guinat, T. Vergne, T. Corre, J.-L. Guérin, M.C. Paul);; Agence Nationale de Sécurité Sanitaire de l’Alimentation, Université Paris-Est, Maisons-Alfort, France (B. Durand);; Direction Générale de l’Alimentation, Paris, France (S. Rautureau);; Agence Nationale de Sécurité Sanitaire de l’Alimentation, Ploufragan, France (A. Scoizec, S. Lebouquin-Leneveu)

**Keywords:** live-duck movements, avian influenza, social network analysis, transmission pathways, animal and public health, France, viruses, influenza, highly pathogenic avian influenza, zoonoses

## Abstract

The relative roles that movement and proximity networks play in the spread of highly pathogenic avian influenza (HPAI) viruses are often unknown during an epidemic, preventing effective control. We used network analysis to explore the devastating epidemic of HPAI A(H5N8) among poultry, in particular ducks, in France during 2016–2017 and to estimate the likely contribution of live-duck movements. Approximately 0.2% of live-duck movements could have been responsible for between-farm transmission events, mostly early during the epidemic. Results also suggest a transmission risk of 35.5% when an infected holding moves flocks to another holding within 14 days before detection. Finally, we found that densely connected groups of holdings with sparse connections between groups overlapped farmer organizations, which represents important knowledge for surveillance design. This study highlights the importance of movement bans in zones affected by HPAI and of understanding transmission routes to develop appropriate HPAI control strategies.

Infectious diseases commonly spread among animal premises by different transmission pathways, including live-animal movement networks that can cause outbreaks in widespread locations or through proximity networks, leading to spatial clusters of outbreaks (*1*,*2*). High-quality data on the spatial distribution of premises have enabled development of transmission models in which the proximity network assumes that any given infectious premises can infect all susceptible premises within a geographic range (*3*,*4*). Increased monitoring of trade-related movement data has enabled the emergence of innovative modeling approaches based on social network analysis (*5*,*6*). Such an approach has been widely used to quantify how animal movement networks have contributed to disease transmission between animal premises (*7*,*8*) and relies on the assumption that premises intensively connected within the network are more likely to become infected and spread infection. Accordingly, efforts have focused on integrating movement and local spread components into models when the dynamics of past epidemics are explored, the effects of control strategies evaluated, and the pattern of future epidemics predicted (*9*–*11*).

However, the relative contribution of movement networks to the overall transmission risks remains poorly understood, compromising assessments of accurate and realistic disease spread modeling and control efforts. First, to assess the likelihood that the infection was acquired from movement networks, tracing of live-animal movement is required but might be challenging, especially in resource-poor settings where movement data are not regularly recorded as part of flock management systems. Then, the order or time at which animal premises become infected must be statistically related to their position in the movement network or in geographic space. However, these dates of infection are often inaccurate because reporting is delayed or completely lacking, particularly when tracking chronic diseases or wildlife populations or when resources are limited.

In 2016–2017, Europe was hit hard by an unprecedented wave of highly pathogenic avian influenza (HPAI) A(H5N8) outbreaks that had severe socioeconomic effects on poultry production, global trade, and human livelihoods (*12*). Most outbreaks were reported in France; ducks were the most affected poultry species (*13*,*14*). The epidemic was contained by the end of March 2017 in France by timely application of measures after detection of the first outbreaks, as provided for by European Union legislation (*15*–*17*). These measures included culling all birds on the infected holdings, establishing of a 3-km protection zone and 10-km surveillance zone with stringent ingoing and outgoing movement bans, testing before movements, and increasing biosecurity measures for holdings in these zones. Spatiotemporal analysis of HPAI outbreaks has shown that the disease spread was partly driven by transmission events between poultry holdings in close proximity in space and time (*14*). Although these previous results helped generate hypotheses about possible routes of infection, they did not enable weighting their relative contribution. Duck movement networks were also identified as underlying factors for the spatial distribution of HPAI outbreaks (*18*), suggesting that these factors should be considered to appropriately describe the epidemic spread. Accurate data on the location and date of suspicion (i.e., onset of clinical signs and increased death rates) of infected holdings and live-duck movements between holdings were collected in France, providing a unique opportunity to unravel the spatial and network dimensions of the epidemic. Our objectives were to analyze live-duck movement networks during the 2016–2017 H5N8 epidemic in France and investigate their likely contribution to disease spread. 

## Methods

### Data Collection

#### Outbreak Data

We obtained data on the H5N8 outbreaks in ducks in France during the 2016–2017 epidemic (November 28, 2016–March 23, 2017) from the Direction Générale de l’Alimentation of the French Ministry of Agriculture (Paris, France). An outbreak was defined as detection of >1 H5N8-infected animal (confirmed by virus isolation or PCR) in a duck holding. Only outbreaks that occurred in holdings that sent or received duck flocks during the study period were retained for the analysis. Data comprised the list of laboratory-confirmed outbreaks, holding identification number, geographic locations (EPSG:2154/RGF93/Lambert-93 [https://epsg.io/2154]), and date of suspicion available by clinical or active surveillance.

#### Trade Movement Data

We considered only duck movements because they represented the most affected poultry species (81.6%) during the epidemic (*14*). The French organization of fattening duck producers (Comité Interprofessionnel des Palmipèdes à Foie Gras [CIFOG]) requires duck producers to report movements from and onto their holdings within 1–2 days of the movement. We thus obtained data on live-duck movements and holdings’ characteristics from the professional database of the CIFOG, under the appropriate confidential data transfer agreements. Data included the list of movement records (defined as movement of a flock between 2 different holdings on the same day), which consisted of the date of movement, identification number of the departure and arrival holdings, and number of ducks moved. The incubation period (i.e., time between virus introduction and onset of clinical signs) ranges from ≈1 to ≈5 days at the individual level and could be longer at the flock level because of the transmission process (*19*). Because such duration is difficult to estimate, a 14-day incubation period was assumed at the flock level (we conducted a sensitivity analysis using a 21-day incubation period and showed that it did not affect the results). Consequently, movements within 14 days before the detection of an infected holding might be responsible for between-holding transmission events (*19*). Thus, we retained only movement data during November 1, 2016–March 31, 2017, between holdings for the analysis; movements to slaughterhouses were excluded. Holdings’ characteristics included the geographic locations (EPSG:2154/RGF93/Lambert-93), group of farmer organization and type of production: rearing (1-day-old ducklings are reared for ≈3 weeks), breeding (1-day to 3-week-old ducks are bred for ≈9–12 weeks), and force-feeding (12-week-old ducks are force-fed for ≈12 days). For holdings with no available coordinates (9.5%), we used the coordinates of the center of the commune (smallest administrative unit in France, with a median area of 10 km^2^).

### Data Analysis

#### Spatiotemporal Description of Movements

We first generated descriptive statistics for the number of active holdings (i.e., holdings that received or sent ducks during the study period), the number of flocks moved, and the distances covered by movements (i.e., using Euclidean distance in kilometers between the departure and arrival holdings) per pair of holdings. We removed holdings without available coordinates from the Euclidean distance estimations. Finally, we mapped the number of movements from/to holdings between departments by aggregating movements at the department level (administrative unit in France corresponding to NUTS [Nomenclature of Territorial Units for Statistics] level 3).

#### Network Analysis

We built directed and weighted networks for data from November 1, 2016–March 31, 2017, considering each duck holding as a node and a movement of a flock between 2 holdings as an edge. We assigned directions to each edge according to the date on which ducks were moved between 2 nodes and assigned weights to each edge according to the number of ducks moved between 2 nodes. We identified trade communities (i.e., densely connected groups of nodes, with only sparse connections between groups [*20*]) over the whole study period using a walktrap algorithm (*21*) based on random walks through the edges in the network. We selected the 15 largest communities on the basis of their respective numbers of holdings and typed them according to holding production types. We mapped holdings belonging to the 15 largest communities and performed a bootstrapped version of the Fisher exact test with 10,000 replicates (*22*) to test whether dependence existed between the trade community and the organization to which farmers belong.

Next, we assessed the likely contribution of live-duck movements in the distribution of H5N8 outbreaks in the network using a permutation-based approach (*23*–*25*). The rationale behind this approach was that if the outbreaks resulted from disease spread through the movement networks, the mean number of infected holdings in contact with an infected holding in the network would be significantly greater than expected if infected holdings were randomly distributed in the network. Again, duck holdings were assumed to become infected through the movement networks if they had received movements from infected holdings within an at-risk period of 14 days before their date of suspicion. Hence, we assumed the mean number of potential transmission events through the movement networks corresponded with the mean number of at-risk movements defined by movements originating from an infected holding (the sender) within 14 days before its date of suspicion and directed to a distinct infected duck holding (the receiver) within 14 days before the receiver’s date of suspicion. We then compared this statistic (i.e., the mean number of transmission events per infected holding) with the distribution of the expected statistic under the null hypothesis according to which the dates of suspicion were randomly distributed among infected holdings in the network (n = 1,000), with the p value corresponding to the proportion of permutations for which the expected statistic is higher than the observed statistic. Similarly, to assess the role of proximity networks, we also conducted the test by calculating the following statistic: the mean number of infected duck holdings close in time (differences of infection dates within 14 days) and space (both located within a 10-km radius [*14*]) per infected duck holding. We selected this space–time window on the basis of previous spatiotemporal analysis conducted on the dataset (*14*). Finally, we identified the likely origins of holding infections by calculating the proportion of infected duck holdings retrieved as receivers in the list of transmission events through the movement network and the proportion of infected duck holdings for which >1 infected duck holding close in time and space was retrieved in the proximity network. On the basis of the movement and proximity networks, we thus attributed to each holding a likely origin of infection as follows: ingoing edge in the movement network only, ingoing edge in the proximity network only, ingoing edges in both movement and proximity networks, and no ingoing edge (i.e., other transmission pathways than by movement and proximity; for example, by introduction of infected migratory birds from northern Eurasia [*13*,*26*]). Because movement bans were reinforced on February 2, 2017 (*27*), we retained only movement and outbreak data for November 1, 2016–February 2, 2017, for this analysis. We conducted all analyses in R statistical software version 3.4.2 using the igraph package (*28*).

## Results

### Spatiotemporal Description

A total of 9,096 movements, involving 10,945,388 ducks moved among 2,098 holdings, occurred during November 1, 2016–March 31, 2017 (Table 1). Most holdings involved in these movements were characterized as force-feeding (48.8%), followed by breeding (35.7%) and breeding plus force-feeding (11.9%). The holdings were located mainly in southwestern and northwestern France (Appendix Figure 1). Overall, most (95.8%) of the flocks were moved from breeding to force-feeding holdings; only 4.2% of movements occurred from rearing to breeding holdings (Table 1). However, more ducks were moved from rearing to breeding holdings (median 4,773) than from breeding to force-feeding holdings (median 958). Movements clearly clustered in the 2 separate geographic areas, southwestern and northwestern France; a limited amount of movements occurred between these 2 areas (Appendix Figure 2).

**Table 1:** Descriptive statistics of duck movements per pair of holdings, France, November 1, 2016–March 31, 2017*

Holding type pair	No. (%) flocks moved	No. ducks moved					Distance moved, km			
		Mean	Median	IQR	Max		Mean	Median	IQR	Max
Rearing to breeding	382 (4.2)	6,001	4,773	3,016–8,991	15,090		58	36	0.1–101	213
Breeding to force-feeding	8,712 (95.8)	993	958	629–1,188	8,050		50	40	16–71	408

*IQR, interquartile range; max, maximum.

### Network Analysis

**Figure 1:**
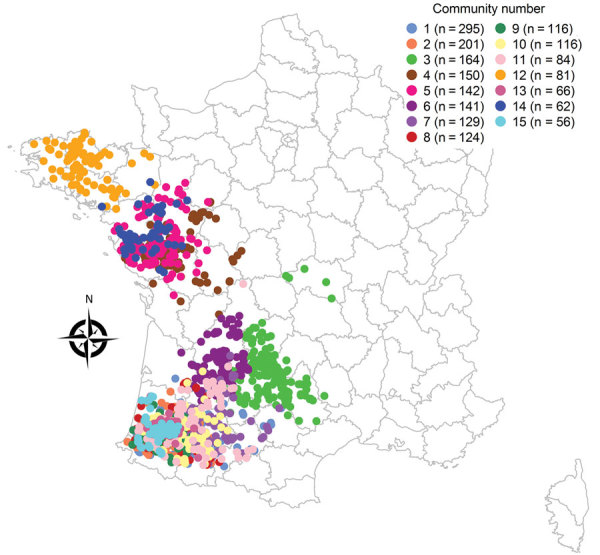
Spatial distribution of the 15 largest live-duck trade communities, France, November 1, 2016–March 31, 2017.

The network analysis identified 99 trade communities comprising 2,098 holdings during November 1, 2016–March 31, 2017. The 15 largest communities in terms of number of holdings included 91.8% of holdings. These communities showed a relatively distinct spatial distribution in northwestern France but completely overlapped in southwestern France (Figure 1). However, the communities were characterized by similar holding compositions, dominated by breeding and force-feeding holdings. The 15 largest communities overlapped significantly with the 15 largest groups of farmer organizations (p<0.001) (Appendix Figure 3): For example, community 1 included 80.0% of holdings belonging to organization A, community 2 included 51.7% of holdings belonging to organization I and 44.8% to organization J, and community 5 included 63.1% and community 12 36.0% of organization B.

**Figure 2:**
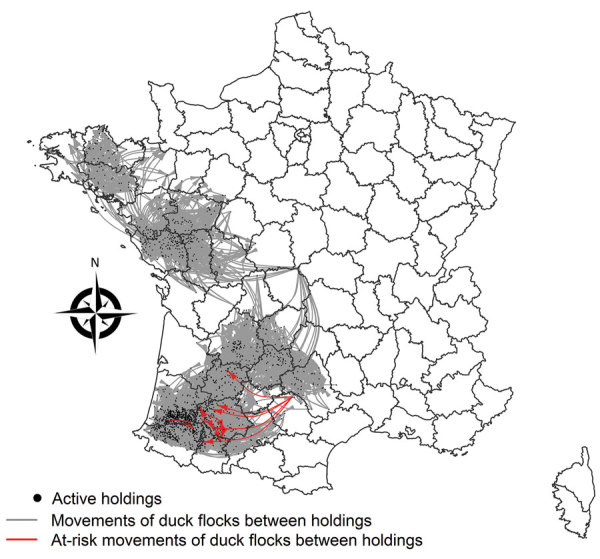
Spatial distribution of live-duck movements identified as responsible for highly pathogenic avian influenza A(H5N8) transmission events between holdings through the movement networks, France, November 1, 2016–February 2, 2017.

A total of 6,521 movements between 1,988 holdings (involving 104 infected holdings) occurred during November 1, 2016–February 2, 2017. Among the 104 infected holdings, 40 (38.5%) were identified as senders, 36 (34.6%) as receivers, and 28 (26.9%) as senders and receivers during that period. Most (989 [91.8%] of 6,521) movements occurred between noninfected holdings. We identified 16 (0.2%) of 6,521 movements as at risk (i.e., they were compatible with transmission events through the movement networks) (Figure 2). These movements mostly occurred between breeding and force-feeding holdings from the end of November through the beginning of January, before stringent movement bans were implemented, and were directed to areas where most outbreaks were reported during the following weeks (Figure 2). Some of the at-risk movements originated from the first outbreak, reported at the beginning of the epidemic (end of November 2016). A few movements (0.4%, 29/6,521) occurred between infected holdings and holdings that did not become infected within 14 days after the movements, from the end of November through the beginning of January, before stringent movement bans were implemented. Therefore, transmission risk through live-duck movements was estimated at 35.5% (16/[16 + 29]), meaning that the likelihood of infection when an infected holding moved flocks to another holding within 14 days before detection was 35.5%. Results from the permutation-based approach indicated the mean number of transmission events per infected holding was significantly greater than under the null hypothesis of an absence of association between movement and infection status (according to which the dates of suspicion should be randomly distributed among network nodes) (p<0.001). Moreover, the mean number of infected holdings close in time and space per infected holding was also significantly greater than expected (p<0.001). By retrieving holding receivers in the list of transmission events through the movement and proximity networks, most sources of holding infection were attributed to proximity networks (66.3%), followed by movement networks (14.4%), and other unknown means of transmission were possible (23.1%) (Table 2). The 16 at-risk movements could be the likely source of infection for only 15 farms because 1 infected farm received 2 at-risk movements.

**Table 2:** Contribution of movement and proximity networks for highly pathogenic avian influenza A(H5N8) transmission events between live-duck holdings, France, November 1, 2016–February 2, 2017

Origin of infection	Infected holdings, no. (%), n = 104	
	14 d before date of suspicion	21 d before date of suspicion
Movement network	11 (10.6)	11 (10.6)
Proximity network	65 (62.5)	72 (69.2)
Movement and proximity networks	4 (3.8)	4 (3.8)
Other	24 (23.1)	17 (16.3)

## Discussion

Using a detailed analysis of live-duck movements and proximity networks, we unraveled the underlying transmission processes of the H5N8 epidemic in ducks in France during 2016–2017. During November 1, 2016–March 31, 2017, which overlaps the H5N8 epidemic period, we observed the most movements from breeding to force-feeding holdings and the largest duck flocks from the rearing to the breeding stage. These findings are consistent with the production cycle and the high specialization in production within which the number of rearing holdings where ducks are first reared and then sent as large flocks to breeding holdings is limited. Flocks are then divided into small flocks to be moved to force-feeding holdings, resulting in a large number of force-feeding holdings reported in the country. Assuming that movement networks influence disease spread, this structure becomes important in terms of disease prevention and control: the dominant role of such superreceiver and superspreader holdings indicates that monitoring only a few holdings would be sufficient to reduce disease spread or that targeting sampling in these high-risk holdings would be more effective than random sampling when time and resources are limited (*29*,*30*). This structure supports the recent active surveillance campaign of duck flocks before movements between these 2 production stages, implemented as a result of the devastating H5N8 epidemic (*31*). Overall, most movements were short range (50% cover <40 km and 75% <75 km). This finding is consistent with results from a spatiotemporal analysis (*14*), which provided evidence that local transmission processes mainly drove the spread. Moreover, our study demonstrated that movements clustered mainly in 2 geographic areas (southwestern and northwestern France) and that a limited number of movements occurred between these 2 areas, potentially explaining why the disease did not spread from south to northwest (*14*).

The 15 largest trade communities that comprised most (91.8%) holdings clearly overlapped with the 15 largest farmer organizations. Again, this finding is crucial in terms of disease surveillance because it highlights that targeting sampling of holdings belonging to the trade community of infected holdings would be more effective than random sampling to prevent further disease spread. In terms of disease control, these results indicate that trade within a given group of highly connected holdings could be maintained by disrupting epidemiologic links to other groups of holdings at risk, mainly to minimize disruption of global trade during an epidemic (*32*,*33*). Moreover, being part of a particular farmer organization implies that holdings are connected by other means than movements of live birds, such as shared transport, equipment, feed, animal staff, or catching teams, that could also facilitate transmission events within the community. Our study also highlighted the important role of the community structure in spreading H5N8: the community to which holdings belong (and thus the farmer organization) was significantly associated with the H5N8 holding infection status (data not shown). Again, trade communities did not overlap between northwestern and southwestern France, which could explain why most of outbreaks remained clustered in southwestern France during the 2016–2017 epidemic (*14*).

Results from our permutation-based approach suggested that a limited proportion of holdings (14.4%) became infected through the movement networks before February 2017. We identified some of these transmission events in the movement networks as originating from the first outbreak reported at the beginning of the epidemic (end of November 2016), before stringent movement bans were implemented, and directed to areas where most of the outbreaks were reported during the following weeks (*14*,*34*). Therefore, despite their low number, live-duck movements might have played a crucial role in the onset and spatial extent of the 2016–2017 H5N8 epidemic in the country. The limited contribution of movement networks to disease spread is most likely explained by the timely implementation of control strategies and movement bans after the first outbreaks were detected (*15*,*16*). This limited contribution also is most likely attributed to the duck production characteristics, highly specialized holdings organized in a small pyramidal structure. Results suggest a transmission risk of 35.5% when an infected holding moves flocks to another holding within 14 days before detection. These findings support efforts by authorities in France in collaboration with the farmer organizations to enhance biosecurity during the transport of ducks (*31*) after successive waves of HPAI outbreaks within 2 years (*14*,*35*). Trucks moving flocks are not allowed to load from several different holdings to minimize the risk for contact infections as trucks travel between holdings. It is likely new rules officials will implement, such as using different sets of trucks and cages to move flocks from breeding to force-feeding and from force-feeding to slaughter. A higher proportion of holdings (66.3%) became infected through proximity networks, consistent with previous work that identified local spread as a predominant transmission pathway in the early stage of the epidemic, that is, before February 2017 (*14*). As a result, these findings also support the national biosecurity program that was implemented to prevent the introduction and spread of poultry diseases at the holding level (*36*,*37*).

The 2 recent devastating epidemics of HPAI in France (2015–2016 and 2016–2017) led to major changes in the collection of movement data. Specifically, the farmer organizations require duck producers to timely and accurately report any details on flock movements, leading to the expectation that underreporting remains limited. Data regarding transport, shared equipment, feed, animal staff, carcass rendering, catching teams, or wild birds were not available (*38*,*39*). However, these transmission pathways might be partly reflected by the proximity network (for example, neighboring holdings might share the same equipment or carcass rendering round) or by 23.1% of holdings for which the infection origin was attributed to transmission pathways other than movement or proximity networks (for example, by introduction of infected migratory birds from northern Eurasia [*13*,*26*]). Although the epidemiologic mechanisms that could explain some of these transmission events remain to be explored, one could infer that these transmission pathways might have played a larger role in the spread of H5N8 between holdings than movement of live ducks. Recent studies have shown that wild birds are likely to have played a minor role in the spread of H5N8 between holdings (*18*,*40*), suggesting that the main driver of the epidemic was holding-to-holding transmission. Further work will compare these results with movement networks during a period with no outbreaks reported as to how outbreaks and intervention strategies have modified the structure of the movement networks.

This study provides insights into the likely contribution of live-duck movement networks into the spread of H5N8 at the beginning of the 2016–2017 epidemic in France. This study also highlights the importance of movement bans in affected zones and that understanding transmission routes is paramount for developing appropriate control strategies for HPAI. A new aspect of this study is the inclusion of a permutation-based approach based on the dates of holding infection to evaluate whether the acquisition of holding infection was consistent with virus transmission through the network. This approach has been limitedly applied in the epidemiology of infectious diseases (*23*–*25*), although it outperforms other degree-based statistical methods, such as logistic regression and nonparametric tests. Outcomes about the relative contribution of movement and proximity networks represent a required basis on which predictive models of HPAI spread could be developed. Finally, this study emphasizes the importance of supplementing epidemiologic data with animal movement data and therefore calls for collaborative efforts to report trade movement data and make them available for appropriately targeting surveillance and interventions during future outbreaks.

Appendix Additional results for study of live-duck movement networks in the transmission of avian Influenza, France, 2016–2017.
