# Supplementary material for: Role of Live-Duck Movement Networks in Transmission of Avian Influenza, France, 2016–2017
Source: Emerg Infect Dis. 2020 Mar;26(3):472–80. doi: 10.3201/eid2603.190412 (PMC7045841; doi:10.3201/eid2603.190412)
Supplement: Appendix — Additional results for study of live-duck movement networks in the transmission of avian Influenza, France, 2016–2017. [file 19-0412-Techapp-s1.pdf]

Article DOI: https://doi.org/10.3201/eid2603.190412

Role of Live-Duck Movement Networks in Transmission of Avian Influenza, France, 2016–2017

Appendix

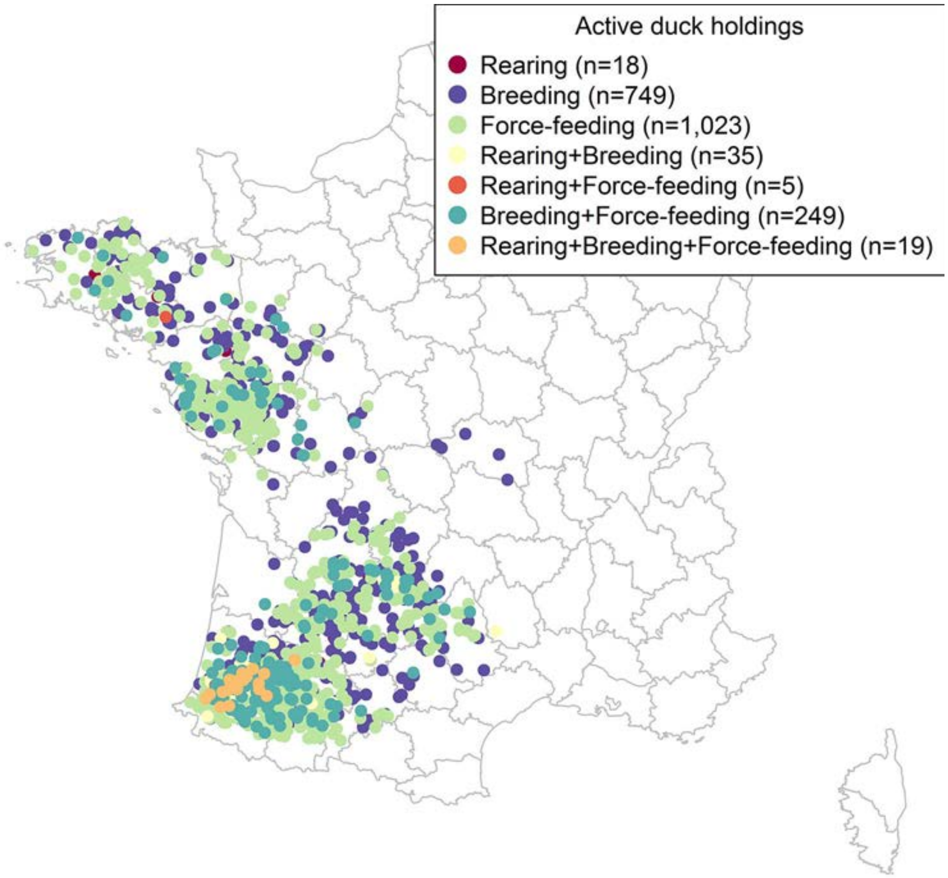

Appendix Figure 1. Spatial distribution of active duck holdings per production type, France, November 1, 2016–March 31, 2017.

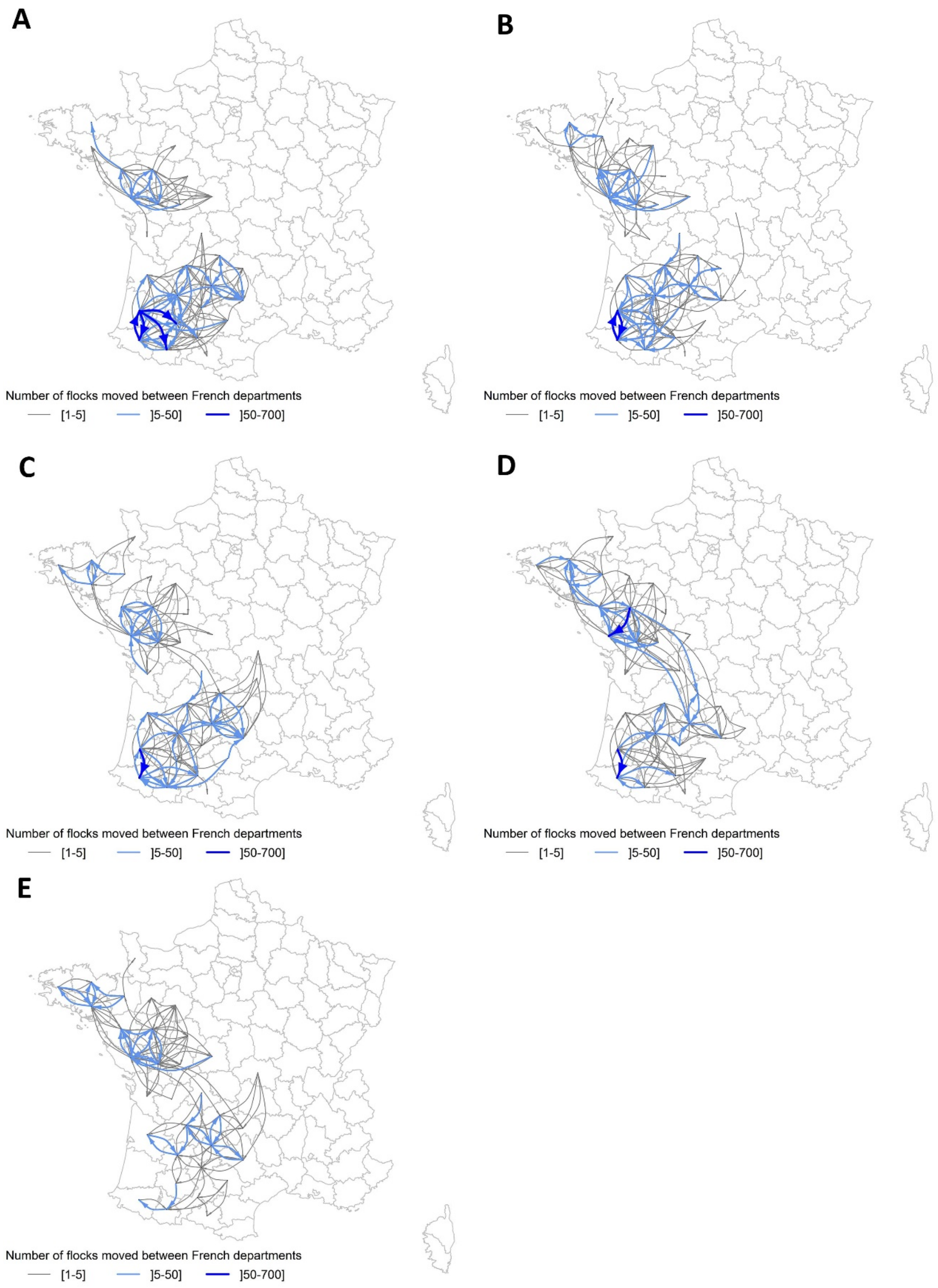

Appendix Figure 2. Spatial distribution of the monthly number of flocks moved between departments, France, November 1, 2016–March 31, 2017. A) November 2016. B) December 2016. C) January 2017. D) February 2017. E) March 2017.

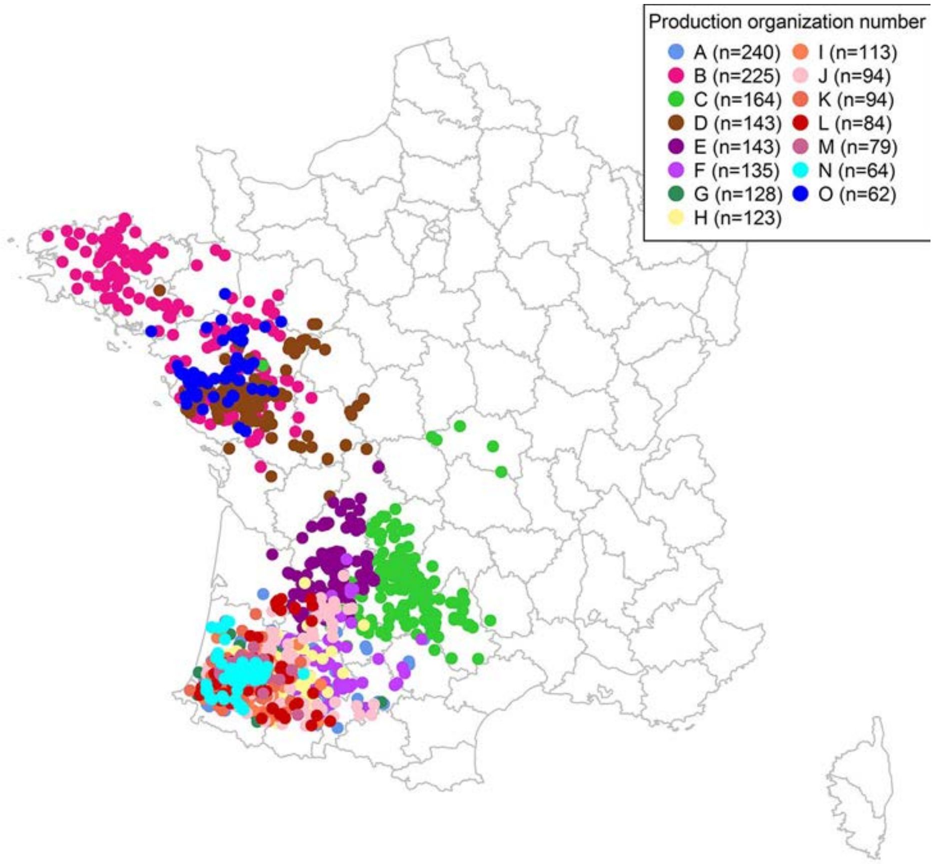

Appendix Figure 3. Spatial distribution of duck holdings per farmer organization type, France, November 1, 2016–March 31, 2017.
